# Collagen Substitutes in Oral Soft Tissue Augmentation: An In Vitro–Ex Vivo Study on Expansion and Elasticity

**DOI:** 10.1002/cre2.70448

**Published:** 2026-09-08

**Authors:** Leonardo Mancini, Stephanie Wenk, Lorenzo Tavelli, Ronald E. Jung, Nadja Naenni, Daniel S. Thoma

**Affiliations:** ^1^ Clinic of Reconstructive Dentistry, Center of Dental Medicine University of Zurich Zurich Switzerland; ^2^ Center for Clinical Research and Evidence Synthesis in Oral Tissue Regeneration (CRITERION) Ann Arbor Michigan USA; ^3^ Department of Oral Medicine, Infection, and Immunity, Division of Periodontology Harvard School of Dental Medicine Boston Massachusetts USA; ^4^ Department of Periodontics and Oral Medicine University of Michigan School of Dentistry Ann Arbor Michigan USA; ^5^ Universidad Catolica de Santiago de Guayaquil (UCSG) Guayaquil Ecuador; ^6^ Department of Periodontology, Research Institute for Periodontal Regeneration Yonsei University College of Dentistry Seoul Korea

**Keywords:** 3D volumetric analysis, blood wetting, collagen matrix, cross‐linked collagen, dental implants, soft tissue substitute

## Abstract

**Objective:**

This study aimed to evaluate the hydration‐induced volumetric expansion and elasticity of collagen‐based soft tissue substitutes following exposure to blood using three‐dimensional (3D) analysis and high‐frequency ultrasonography (HFUS).

**Materials and Methods:**

Seven commercially available collagen matrices were analyzed: Fibro‐Gide (6 mm and 3 mm), Mucogain (5 mm and 3 mm), Volumax, Collagen Graft II, and Mucograft. The investigation consisted of two experimental phases. In the in vitro phase, samples were immersed in stabilized porcine blood to assess absorption index (AI), 3D volumetric changes, and ultrasonographic (HFUS) parameters, including density and elasticity. In the ex vivo phase, matrices were placed beneath envelope flaps in porcine maxillae, with optical 3D scanning performed before and after blood saturation. Statistical analysis included generalized estimating equations for repeated measures and nonparametric testing, with significance set at *α* = 0.05.

**Results:**

Volume stable matrices exhibited significantly greater absorption and volumetric expansion compared to non‐stable matrices. Fibro‐Gide 6 mm demonstrated the highest absorption index (17.0 ± 0.6 mg) and volumetric increase (268.4 ± 54.8 mm^3^), while Mucogain 5 mm showed the greatest vertical expansion (0.83 ± 0.08 mm). Non‐stable matrices displayed reduced expansion or partial collapse. A positive, though non‐significant, correlation was observed between density (HFUS) and volumetric expansion (*r* = 0.29, *p* = 0.535).

**Conclusions:**

Volume‐stable collagen matrices show pronounced fluid uptake and expansion. Clinically, this effect should be considered, as excessive swelling may increase flap tension and compromise wound stability, necessitating careful trimming and adequate flap release.

## Summary

**What Is Already Known on This Topic:**

∘ Collagen‐based soft tissue substitutes are widely used as alternatives to autogenous grafts in peri‐implant soft tissue augmentation.

∘ Their clinical performance depends on biological integration and physical properties such as hydration and structural stability.

∘ Early volumetric changes after blood exposure remain insufficiently quantified.

**What This Study Adds:**

∘ Volume‐stable collagen matrices show significantly greater blood absorption and 3D volumetric expansion compared to non‐volume stable matrices.

∘ Soft tissue substitute thickness has an influence on primary wound closure.

∘ The magnitude and pattern of expansion may influence flap tension and surgical outcomes, emphasizing the need for material‐specific clinical handling.

## 1 Introduction

Soft tissue augmentation around dental implants plays a crucial role in maintaining long‐term peri‐implant health and mucosal stability, as well as in enhancing esthetics. Sufficient horizontal and vertical mucosal thickness (> 2 mm) has been associated with reduced marginal bone loss and improved esthetic outcomes (Berglundh and Lindhe 1996; Linkevicius et al. 2009; Tavelli, Barootchi, Avila‐Ortiz et al. 2021). Traditionally, autogenous connective tissue grafts (CTGs) harvested from the palate have been considered the gold standard due to their predictable biological integration and stability (Stefanini et al. 2023; Thoma et al. 2014). However, CTG harvesting is associated with donor‐site morbidity, limited availability, and extended surgical time, motivating the development of xenogeneic soft tissue substitutes (Stefanini et al. 2021; Thoma, Strauss et al. 2023).

Collagen‐based matrices have emerged as reliable alternatives for peri‐implant soft tissue thickening. These matrices, derived mainly from porcine or bovine collagen, can be cross‐linked or non–cross‐linked, a factor that significantly influences their biomechanical and biological behavior (Schmitt et al. 2016; Thoma et al. 2012). Cross‐linking enhances matrix stability and slows degradation, thereby improving handling and volume maintenance. However, it may also reduce porosity and cellular infiltration (Thoma et al. 2012; Bienz et al. 2023; Song et al. 2019; Thoma et al. 2017). Non–cross‐linked matrices, conversely, exhibit greater hydrophilicity and biological integration but are prone to rapid resorption (Mancini, Romandini et al. 2021; Yao et al. 2008).

The hydration characteristics of collagen matrices influence the in situ volumetric behavior, particularly during early healing when they are exposed to blood and tissue fluids. Excessive expansion may alter flap tension, impair wound closure, or distort the intended soft tissue contours (Thoma et al. 2012; Bienz et al. 2023; Yao et al. 2008; Thoma, Gasser et al. 2023). Conversely, moderate swelling can promote rapid clot stabilization and cellular migration. While previous studies have described the hydration capacity of various collagen scaffolds qualitatively or via linear measurements only on clinical scenarios, quantitative analysis, absorption index, three‐dimensional (3D) analysis of volumetric expansion, elasticity, and density after blood exposure remains limited (Thoma et al. 2017; Mancini, Khehra et al. 2023; Tavelli et al. 2022). Therefore, the present in vitro study aims to analyze the different characteristics and mainly the volumetric expansion of seven commercially available collagen‐based soft tissue substitutes after immersion in pig blood using 3D scanning analysis, absorption index (AI), and high‐frequency ultrasonography. We hypothesize that volume‐stable matrices will exhibit significantly higher volumetric expansion than non‐volume stable matrices.

## 2 Materials and Methods

**Figure 1:**
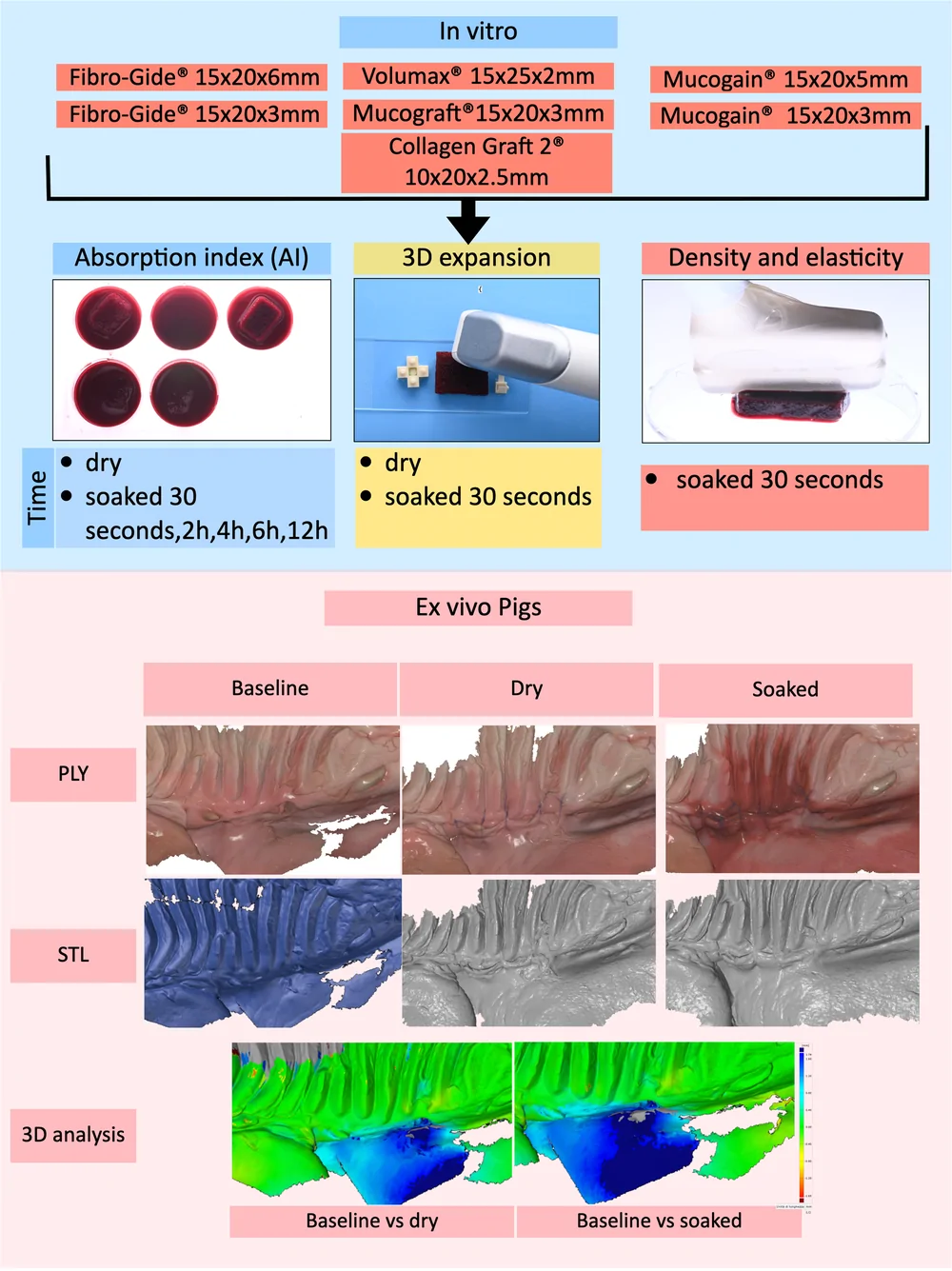
Phase I (in vitro): Evaluation of absorption index (AI), volumetric changes, density, and elasticity of seven soft tissue matrices after blood soaking. Phase II (ex vivo): 3D analysis on porcine jaws assessing collagen matrix expansion beneath the flap before and after blood saturation. PLY, polygon file format; STL, standard tessellation language.

This study was conducted at the Clinic for Reconstructive Dentistry, University of Zurich, following the CRIS (Checklist for Reporting In‐vitro Studies) guidelines (Krithikadatta et al. 2014). Ethical approval was not required for this study as it did not involve human or animal subjects. Blood samples and pig jaws were obtained from a local butcher and had not been previously frozen or otherwise altered. Figure 1 illustrates the soft tissue substitutes involved, outcomes measured, the time points for data collection, and the different steps of the study.

### 2.1 Study Design

The study was designed in two phases. The first phase consisted of an in vitro analysis evaluating the absorption index (AI), volumetric changes, density, and elasticity of seven different soft tissue matrices following blood soaking. The second phase was conducted ex vivo using porcine jaws, where three‐dimensional (3D) analysis was performed to investigate the expansion and behavior of the collagen matrix beneath the surgical flap before and after blood saturation.

### 2.2 Materials Tested

Details on each soft tissue substitute are summarized in Table 1.

**Table 1:** Collagen‐based soft tissue substitutes used in the study and their characteristics, dimensions, and structural features.

Matrix	Manufacturer	Cross‐linking	Composition	Structure	Size	Code
Fibro‐Gide	Geistlich Pharma, Switzerland	Cross‐linked. Smart cross‐linking.	Porcine collagen type I and III	Volume stable 3D Matrix	*N* (15) 15 × 20 × 6 mm *N* (15) 15 × 20 × 3 mm	FG6FG3
Mucogain	Nobel Biocare, AB, Gothenburg, Sweden; previously Mucomaix, Matricel GmbH, Germany	Non‐cross‐linked	Porcine collagen type I and III	Volume stable 3D Matrix	*N* (15) 15 × 20 × 5 mm *N* (15) 15 × 20 × 3 mm	MG5MG3
Volumax	Dentsply, Germany	Cross‐linked. Sugar cross‐linking via GLYMATRIX	Porcine collagen type I	Volume stable 3D Matrix	*N* (15) 15 × 25 × 2 mm	VC
Collagen Graft II	Genoss, South Korea	Cross‐linked	Porcine collagen type I	2 layers: compact and spongious	*N* (15) 10 × 20 × 2.5 mm	CG2
Mucograft	Geistlich Pharma, Switzerland	Non–cross‐linked	Porcine collagen type I and III	2 layers: compact and spongious	*N* (15) 15 × 20 × 3 mm	MC

### 2.3 Phase I (In Vitro)

#### 2.3.1 Sample Preparation

Matrices were used in their original fabricated size and shape without further modification. Samples were weighed (dry) and stored in sterile Petri dishes prior to testing (*n* = 5 per group).

#### 2.3.2 Blood Soaking Protocol and Absorption Index (AI)

Fresh porcine blood was collected from a local butcher and stabilized with sodium citrate 3.8% (Amino AG, Gebenstorf, Switzerland) with a ratio of 1:9 mL, meaning 222 mL per 2 L of blood to prevent coagulationFive samples for each substitute were individually immersed in a Petri dish (55 mm × 14 mm) with 20 mL of blood at 37°C (Elemental Kletter, Elemental, Ghent, Belgium). The samples were weighed (Mettler AT261 Delta Range, Mettler‐Toledo, Switzerland) prior to and following the removal of the blood at different time points: Dry (T0), 30 s (T1), 2 h (T2), 4 h (T4), 6 h (T6), and 12 h (T12). The difference in mass was used to calculate the absorption index (AI) for each material in milligrams (mg) at different time points.

AI=(Wwet(mg)−Wdry(mg))/Wdry(mg)

#### 2.3.3 3D Scanning and Volumetric Analysis

Samples were scanned before and after soaking using an intraoral 3D scanner (TRIOS 3, 3Shape, Copenhagen, Denmark). The resulting STL files were analyzed using 3D metrology software (Gom Inspect 2019, Carl Zeiss AG, Germany). Volumetric changes and mean dimensional variations (thickness, mm) were calculated through superimposition and volumetric comparison. Expansion or contraction on the vertical and horizontal axes was also recorded to highlight different deformation patterns associated with the various types and thicknesses of the substitutes.

To standardize the procedure and minimize potential volumetric variations between the dry and wet phases, a microscope slide equipped with two customized scanning pins was used. The different substitutes were positioned at the center of the slide and scanned before and after wetting. Wetting was performed by applying porcine blood with a syringe for 30 s. If necessary, excess blood was removed before the samples were scanned. The detailed steps of the scanning procedure are illustrated in Figure S1.

#### 2.3.4 High‐Frequency Ultrasonography Evaluation of Density and Elasticity

Ultrasonographic evaluation of the seven different matrices was performed using a high‐frequency ultrasound (HFUS) probe (Esaote MyLab, Esaote S.p.A., Genoa, Italy). Images were recorded and subsequently analyzed using ImageJ software (ImageJ, National Institutes of Health, Bethesda, MD) to evaluate grayscale level matrices mean pixel/echo intensity (EI). The detailed protocol for image acquisition and analysis has been previously described in another study (Mancini, Khehra et al. 2023). In addition, 5‐s video clips were recorded to assess matrix elasticity a dedicated software (US‐Elasto, Dileny Technologies, Giza, Egypt) capable of computing frame‐to‐frame displacement and superimposing strain values on B‐mode images as a color‐coded elastogram. Tissue stiffness was quantified as a frame‐to‐frame strain ratio (SR) reflecting the stiffness of the matrix relative to a non‐deformable reference, as previously described (Mancini, Khehra et al. 2023; Tavelli and Barootchi 2024). The closer an SR approaches 1, the higher the stiffness/rigidity of the matrix; conversely, an SR approaching 0 indicates a relatively low stiffness/rigidity and high strain/elasticity. Cine loops were acquired during a controlled freehand quasi‐static compression applied with the transducer. Strain elastography was expressed as a strain ratio between two predefined regions of interest (ROIs) within the same cine loop, as previously described (Tavelli and Barootchi 2024). Because both ROIs were subjected to the identical compression, the absolute magnitude of the applied force cancels out in the ratio calculation, allowing the measurement to reflect the relative mechanical properties of the tissues rather than the compression force itself (Tavelli and Barootchi 2024).

### 2.4 Phase II (Ex Vivo)

The study was conducted on both sides of the maxillae. Prior to any surgical intervention, optical intraoral impressions of the entire maxillary arch were obtained using an intraoral scanner (TRIOS 3; 3Shape, Copenhagen, Denmark). A total of 35 sample sites were prepared, including five sites per collagen matrix.

Each experimental site within the same subject was consecutively allocated to one of the seven collagen matrices. The surgical area comprised the edentulous region between the lateral incisor and the canine on both sides of the maxilla (Figure 1).

Sulcular incisions were performed at the adjacent teeth, followed by a crestal full‐thickness incision connecting the palatal line angles. A buccal split‐thickness flap was then prepared, extending from the mesial line angle of the incisor to the distal line angle of the canine. On the palatal aspect, the assigned collagen matrix was positioned and stabilized using a modified horizontal mattress suture. Primary wound closure was subsequently achieved with interrupted simple sutures (Dafilon 6‐0, B. Braun, Melsungen, Germany).

After placement of the matrices in a dry condition, an additional intraoral scan was obtained. The flap was then carefully reopened only at the central portion of the incision, avoiding mobilization of the mesial and distal flap margins to ensure accurate repositioning. Previously stabilized porcine blood (5 mL), prepared as described above, was injected into the matrices over 30 s until saturation was achieved. Saturation was defined as the point at which excess blood began to emerge from the injection site. The excess blood was removed by suction, with care taken to restrict suction to the flap surface and avoid direct contact with the matrices. The suture removed to permit injection was subsequently replaced in its original position to restore complete wound closure.

**Figure 2:**
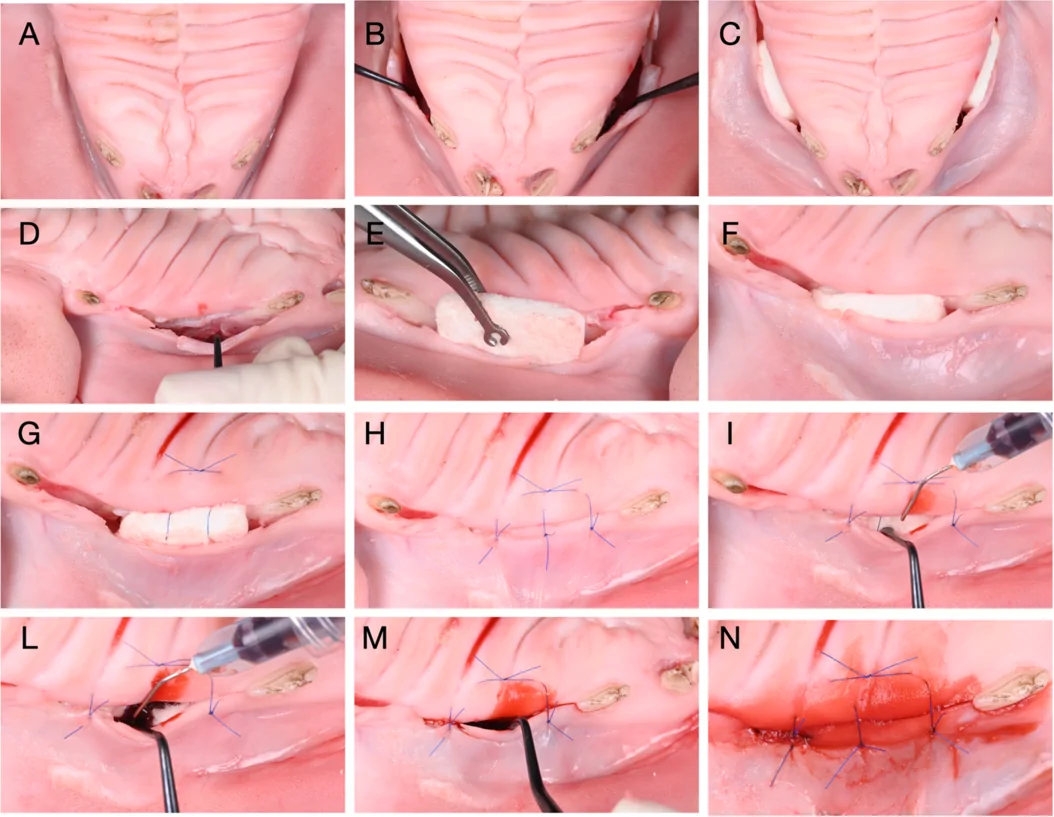
(A) Baseline clinical situation. (B) Envelope flap design. (C–F) Placement of the soft tissue substitute. (G) Palatal fixation of the matrices. (H) Primary wound closure using simple interrupted sutures. (I–M) Removal of the central suture followed by injection of stabilized blood into the surgical site. (N) Primary wound closure after repositioning the central suture at the same flap location.

Following final suturing, a subsequent intraoral scan (Trios; 3Shape, Copenhagen, Denmark) was performed. This procedure was repeated five times for each matrix type (Figure 2).

Digital models were acquired using an intraoral optical scanner (Trios; 3Shape, Copenhagen, Denmark) and subsequently imported into dedicated image analysis software (GOM Inspect; GOM GmbH, Braunschweig, Germany). All measurements were conducted by an experienced examiner. Using predefined, reproducible reference points and a best‐fit alignment algorithm, a semi‐automated superimposition protocol was implemented, as previously described in the literature (Borges et al. 2020; Parvini et al. 2021). The baseline model served as the reference dataset and was superimposed onto each corresponding model representing the different increments of the seven soft tissue substitutes before and after soaking (Mancini, Fratini et al. 2021). A standardized region of interest (ROI) was delineated in accordance with established volumetric assessment protocols (Tavelli et al. 2022; Mancini et al. 2024, Mancini, Galarraga‐Vinueza 2023; Zuercher et al. 2023). Volumetric outcomes included (i) total volume change (Vol) and (ii) the mean surface distance between superimposed models, representing the mean thickness of the augmented tissue. These parameters were calculated following previously validated methodologies (Schmitt et al. 2016; Fons‐Badal et al. 2020; Tavelli, Barootchi, Majzoub et al. 2021; Tian et al. 2019).

### 2.5 Statistical Analysis

The sample included *n* = 35 matrices in a balanced design of 7 different. A test of absorption was performed at time points T0 (dry), T1 (30 s soaked), T2 (2 h), T4 (4 h), T6 (6 h), and T12 (12 h), for measuring the outcome absorption index (AI).

Statistical analysis consists of a descriptive of all continuous parameters (mean, standard deviation, range, median, and quartiles). The absorption index was analyzed using linear regression with generalized estimation equations (GEE) with group and timepoint as independent factors. Main effects and interactions were estimated from Wald's Chi2 statistic using an exchangeable correlation matrix with robust Huber–White estimators. This methodology was chosen to control the within‐specimen dependence of repeated measurements over time, providing best fit than unstructured matrix correlation. Multiple pairwise comparisons were conducted using Bonferroni's test.

Parameters of the 3D in vitro analysis and US density analysis were analyzed using the Kruskal–Wallis test for the overall between‐groups comparison and Mann–Whitney's test corrected by Bonferroni for multiple pairwise comparisons. The significance level used in the analysis was 5% (*α* = 0.05).

Mean gray levels per matrix type were calculated from the HFUS (high‐frequency ultrasonography) data by averaging seven values. For the ex vivo data, mean volumetric expansion per matrix type was calculated from seven values for each comparison (baseline–dry, baseline–wet). Spearman's correlation analysis was then performed using the newly aggregated dataset comprising seven cases.

## 3 Results

### 3.1 Weight Increase and Absorption Index (AI)

The seven matrix types analyzed demonstrated distinct and time‐dependent weight variation profiles following immersion.

Fibro‐Gide 6 mm (FG6) exhibited the most pronounced increase immediately after soaking (T1), with a mean weight change of 1958.1 ± 164.8 mg, followed by subsequent stabilization over time. Mucogain 5 mm (MG5) showed an intermediate increase at T1 (1118.7 ± 192 mg), with a continued progressive rise throughout the observation period. In contrast, Volumax (VC) demonstrated only a modest but steady increase, reaching a mean weight of 233.5 ± 21.5 mg at T1 (Figure S2).

Intergroup comparisons revealed statistically significant differences at multiple time points. At T1 (after soaking) and T2 (2 h), the absorption index value of FG6 was significantly higher than that of all other matrices (*p* < 0.001). At T4 (4 h), FG6 remained significantly higher than all groups except MG5, where marginal significance was observed (*p* = 0.075). At T6 (6 h), FG6 again showed significantly higher index values compared to all other matrices. By T12 (12 h), FG6 maintained significantly higher values than all groups except MG5 (*p* = 0.521). Furthermore, MG5 exhibited significantly higher index values than all other matrices except Mucograft (MC), where marginal significance was noted (*p* = 0.060).

The intragroup analysis demonstrated Fibro‐Gide 3 mm (FG3) to be the most dimensionally stable matrix, with no statistically significant difference between T1 (post‐soaking) and T12 (12 h) measurements. In contrast, the remaining collagen matrices exhibited a progressive weight increase from T1 to T12, although the rate of increase varied among materials, reflecting distinct absorption kinetics and structural behaviors.

Figure S2 shows the mean weights at different time points, while Table S1 summarizes the corresponding absorption index (AI) values. Overall, AI after soaking remained stable over time, except for Mucogain 3 mm (MG3), which showed an increase from 17.15 ± 2.74 mg at T1 (post‐soaking) to 21.98 ± 1.28 mg at T12 (12 h). Volumax (VC) exhibited a similar increasing trend, with AI values of 4.14 ± 0.47 mg at T1 and 11.33 ± 1.78 mg at T12.

### 3.2 3D Scanning and Volumetric Analysis

#### 3.2.1 3D Mean

The 3D analysis showed different expansion pattern (*p* < 0.001) according to the 7 substitutes analyzed, regarding the 3D mean dimensional variation FG6 (0.48 ± 0.06 mm), MG5 and MG3 and CG2 (0.53 ± 0.40 mm; 0.40 ± 0.12 mm; 0.46 ± 0.11 mm) showed the highest distribution of overall mean compared to FG3 (0.21 ± 0.07 mm), VC(−0.28 ± 0.23 mm) and MC (−0.70 ± 0.10 mm).

#### 3.2.2 3D Volume

**Figure 3:**
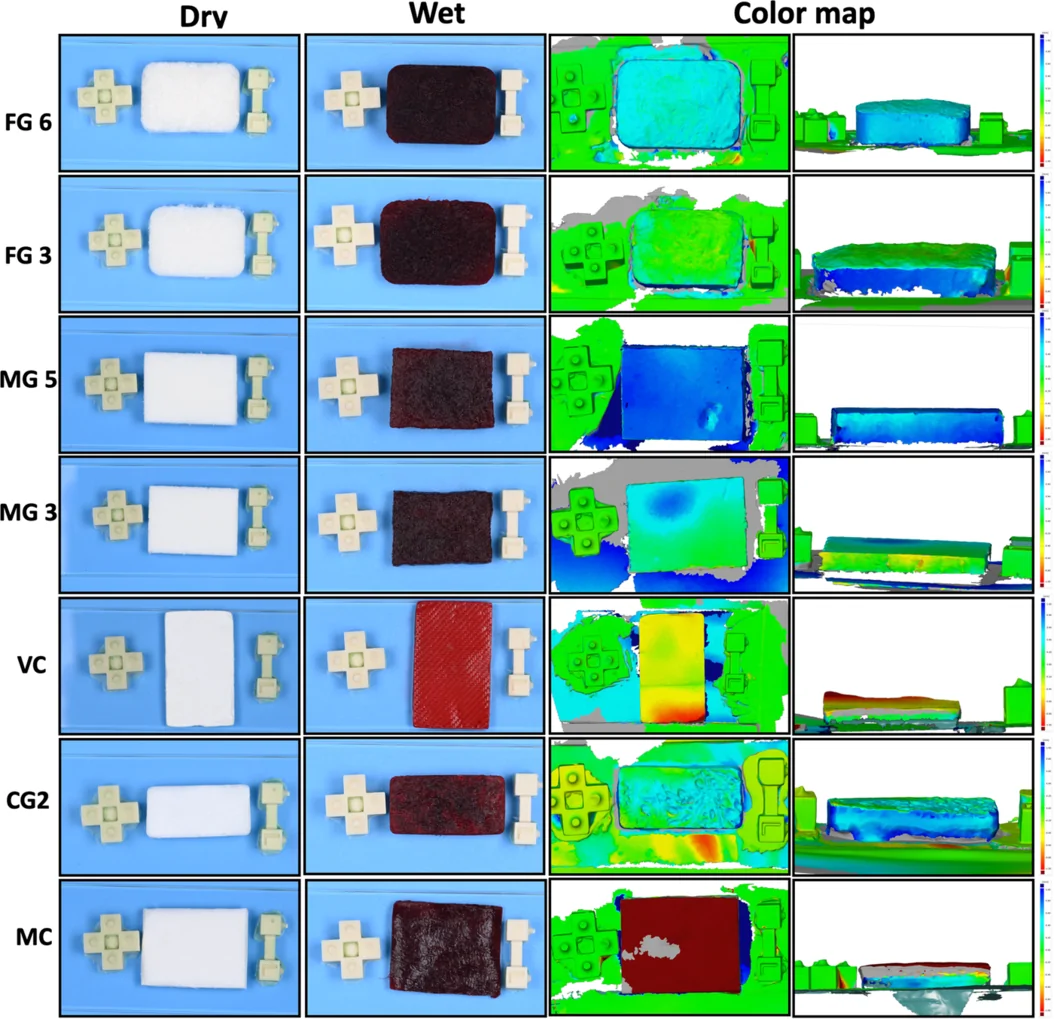
Representative illustrations of the different materials evaluated under dry conditions and after 30 s of soaking. Occlusal and lateral views illustrate the dimensional changes observed, where blue indicates expansion, red indicates collapse, and green indicates dimensional stability. (FG6) Fibro‐Gide6 mm; (FG3) Fibro‐Gide 3 mm; (MG5) Mucogain 5 mm; (MG3) Mucogain 3 mm; (VC), Volumax; (CG2) Collagen Graft 2; (MC) Mucograft.

The overall 3D volumetric analysis revealed variations according to material and thickness. FG6 and MG5 exhibited the greatest volume gains (268.4 ± 54.8 mm^3^ and 371.0 ± 75.1 mm^3^, respectively), whereas MC showed the greatest volume loss (−363.5 ± 80.5 mm^3^). FG3, MG3, and CG2 demonstrated volume gains of 96.7 ± 33.5 mm^3^, 191.2 ± 60.8 mm^3^, and 156.1 ± 32.9 mm^3^, respectively, while VC exhibited a volume loss of −127 ± 3 mm^3^. Figure 3 provides a clear visualization of these changes using a color‐coded map: blue indicates volume gain, red indicates volume loss, and green indicates stability. Compared with the dry‐condition baseline, volume increased by 18.2% for FG6, 10.2% for FG3, 28.6% for MG5, 26.2% for MG3, 34.5% for CG2, whereas VC and MC showed a decrease of −20.9% and −57.7%.

#### 3.2.3 3D Vertical Expansion Pattern

On the vertical aspect, the rebound effect of the different substitutes demonstrated a range of expansion that differed significantly among the groups (*p* < 0.001). MG5 exhibited the greatest increase (0.83 ± 0.08 mm), followed by MG3 and CG2 (0.44 ± 0.16 mm and 0.44 ± 0.14 mm, respectively), and FG6 (0.33 ± 0.04 mm). In contrast, FG3 showed occlusal stability (0.03 ± 0.07 mm), while MG and VC exhibited collapse, with reductions of −1.11 ± 0.24 mm and −0.37 ± 0.28 mm, respectively.

#### 3.2.4 3D Horizontal Expansion Pattern

**Figure 4:**
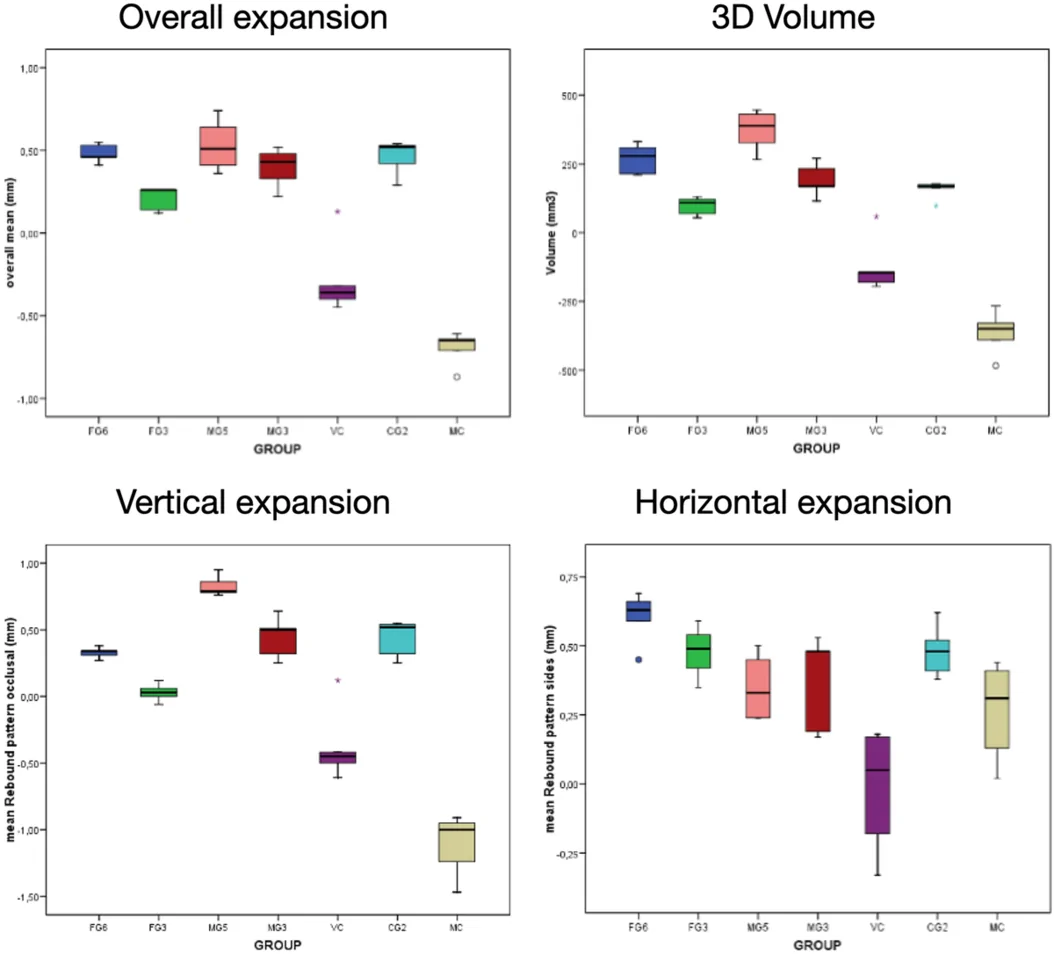
Box plots from the 3D analysis illustrated the overall expansion, volume increase, and volume expansion at the occlusal and lateral sites. Notably, MG5 and FG6 demonstrated greater expansion and volume increase compared with the other soft tissue substitutes. (FG6) Fibro‐Gide 6 mm; (FG3) Fibro‐Gide 3 mm; (MG5) Mucogain 5 mm; (MG3) Mucogain 3 mm; (VC), Volumax; (CG2) Collagen Graft 2; (MC) Mucograft.

On the lateral sides of the different matrices, the rebound effect demonstrated a pattern distinct from that observed on the occlusal aspect. FG6 exhibited the greatest expansion (0.60 ± 0.09 mm), followed by FG3 and CG2 (0.48 ± 0.10 mm and 0.48 ± 0.09 mm, respectively). MG3 and MG5 showed moderate expansion (0.37 ± 0.17 mm and 0.35 ± 012 mm, respectively), whereas MC demonstrated a smaller expansion (0.26 ± 0.18 mm). In contrast, VC showed minimal dimensional change, with a slight shrinkage (−0.02 ± 0.23 mm) Figure 4.

### 3.3 High‐Frequency Ultrasonography Evaluation of Density and Elasticity

#### 3.3.1 Mean Gray Level

The mean pixel echo intensity (EI) showed different distributions among the seven evaluated substitutes (*p* < 0.025). FG6 exhibited the lowest EI (62.82 ± 5.99 GL), followed by FG3 (70.32 ± 19.31 GL), MC (73.05 ± 13.92 GL), CG2 (85.44 ± 11.19 GL), and VC (86.57 ± 9.06 GL). The highest EI values were observed for MG3 and MG5, with means of 90.60 ± 36.47 and 105.36 ± 6.24 GL, respectively.

#### 3.3.2 Skewness and Kurtosis

Skewness and kurtosis differed significantly among the groups, with statistically significant differences in their distributions (*p* = 0.007 and *p* = 0.002, respectively). FG6 exhibited the highest skewness compared to VC and MG5, which showed the lowest values. A similar trend was observed for kurtosis, with FG6 presenting the highest distribution, whereas VC and MG5 showed lower values (Table S2).

#### 3.3.3 Elastography

**Figure 5:**
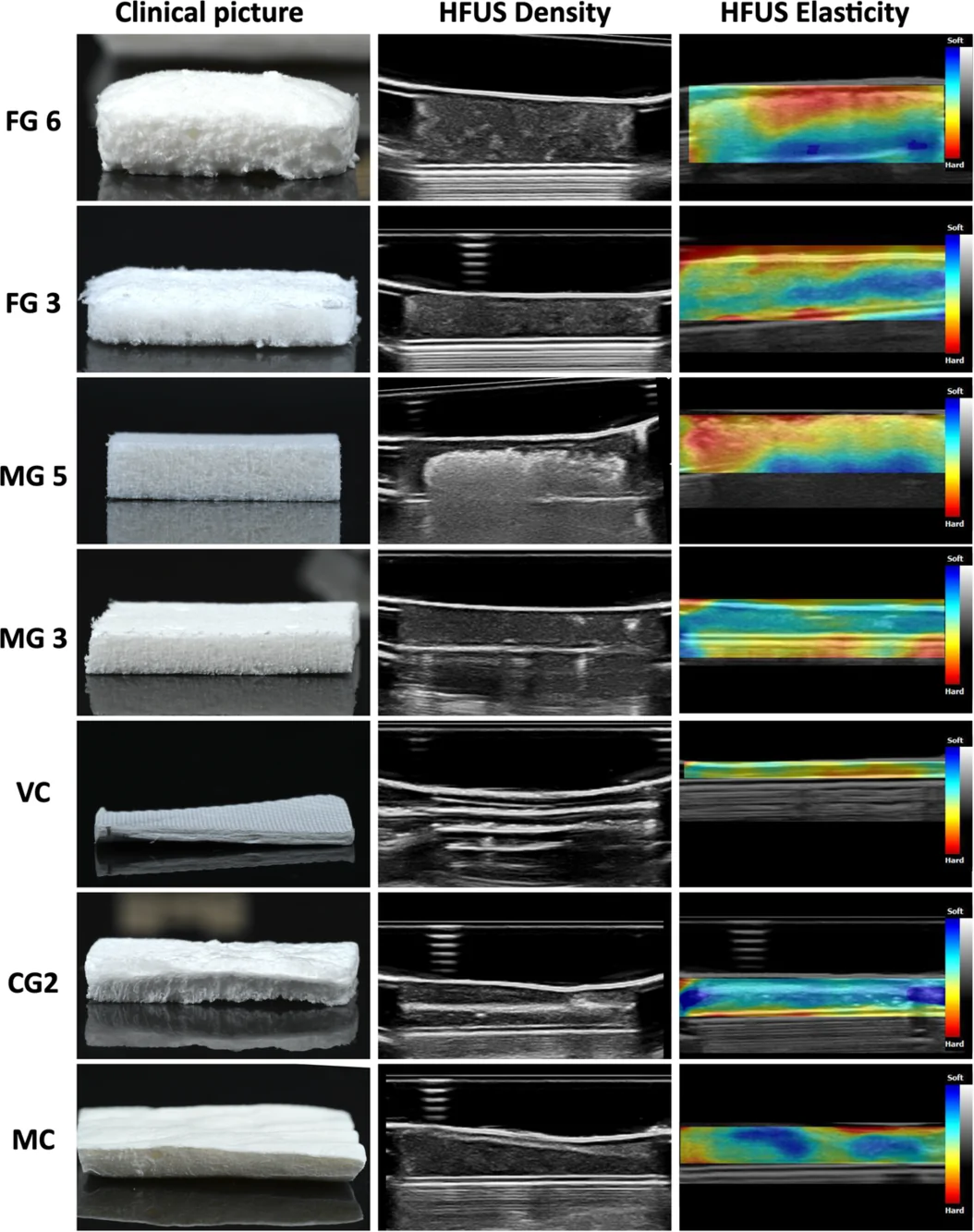
Representation of the different soft tissue substitutes, along with their ultrasonographic density and elasticity. (FG6) Fibro‐Gide 6 mm; (FG3) Fibro‐Gide 3 mm; (MG5) Mucogain 5 mm; (MG3) Mucogain 3 mm; (VC) Volumax; (CG2) Collagen Graft 2; (MC) Mucograft. The HFUS density showed, through the gray level scale, hypo‐ and hyperechoic regions, low gray levels indicating a low density of the material, while high gray scale showing a higher density. The elasticity showed clearly that during compression, the behavior of the different collagen matrices had different type of density and portion with hard and soft component.

The elastographic analysis based on SRs demonstrated that VC had the highest stiffness/rigidity compared to the other materials (*p* < 0.0001) with a SR value of 0.47 ± 0.08 SR; followed by FG6 0.38 ± 0.10, FG3 0.32 ± 0.06, MG5 0.29 ± 0.10; MG3 0.23 ± 0.04, and with the least SR value CG2 0.18 ± 0.02 and MC 0.19 ± 0.05. Figure 5 shows the high‐frequency ultrasonography and elasticity of the different materials.

### 3.4 Ex Vivo 3D Analysis

#### 3.4.1 Baseline versus Dry Matrices

The overall mean surface deviation indicated that FG6 and MG5 exhibited the greatest expansion, with mean values of 4.40 ± 0.88 mm and 3.42 ± 0.61 mm, respectively. These were followed by FG3 (3.28 ± 0.54 mm), MG3 (2.24 ± 0.36 mm), CG2 (2.25 ± 0.30 mm), VC (2.04 ± 0.59 mm), and MC (1.92 ± 0.59 mm).

Similarly, the volumetric gain analysis demonstrated the highest increase for FG6 (701.33 ± 138.15 mm^3^), followed by MG5 (519.09 ± 93.14 mm^3^) and FG3 (505.72 ± 81.99 mm^3^). Lower volumetric increases were observed for CG2 (339.80 ± 47.24 mm^3^) and MG3 (329.11 ± 58.11 mm^3^), while VC (296.30 ± 81.71 mm^3^) and MC (294.58 ± 88.78 mm^3^) showed the smallest volumetric changes.

#### 3.4.2 Baseline versus Soaked Matrices

The analysis of overall mean surface deviation showed that FG6 and FG3 exhibited the greatest expansion, with mean values of 4.81 ± 0.59 mm and 3.50 ± 0.51 mm, respectively. These were followed by MG5 (3.42 ± 0.64 mm), VC (2.41 ± 0.34 mm), MG3 (2.35 ± 0.28 mm), CG2 (2.24 ± 0.28 mm), and MC (1.29 ± 0.46 mm).

Consistent with these findings, the volumetric gain analysis revealed the highest increase for FG6 (754.44 ± 108.58 mm^3^), followed by FG3 (535.56 ± 80.56 mm^3^) and MG5 (524.45 ± 99.32 mm^3^). Intermediate volumetric increases were observed for VC (359.68 ± 35.87 mm^3^) and MG3 (347.12 ± 36.85 mm^3^), whereas CG2 (338.35 ± 41.25 mm^3^) and MC (202.64 ± 72.19 mm^3^) exhibited the lowest volumetric changes.

#### 3.4.3 Comparison Between Dry and Soaked Matrices 3D Analysis

A comparison between the dry and soaked matrices demonstrated a general increase in both surface deviation and volumetric gain following hydration. FG6 showed a noticeable increase in mean surface deviation from 4.40 ± 0.88 mm (dry) to 4.81 ± 0.59 mm (soaked), accompanied by a rise in volumetric gain from 701.33 ± 138.15 mm^3^ to 754.44 ± 108.58 mm^3^. Similarly, FG3 exhibited an increase in both parameters, with surface deviation increasing from 3.28 ± 0.54 mm to 3.50 ± 0.51 mm, and volumetric gain from 505.72 ± 81.99 mm^3^ to 535.56 ± 80.56 mm^3^ after soaking.

In contrast, MG5 showed comparable dimensional expansion between conditions, with mean surface deviation remaining stable (3.42 ± 0.61 mm dry vs. 3.42 ± 0.64 mm soaked), although a slight increase in volumetric gain was observed (519.09 ± 93.14 mm^3^ to 524.45 ± 99.32 mm^3^). Moderate increases were also observed for VC, where surface deviation increased from 2.04 ± 0.59 mm to 2.41 ± 0.34 mm, and volumetric gain from 296.30 ± 81.71 mm^3^ to 359.68 ± 35.87 mm^3^.

Conversely, MG3 and CG2 showed minimal changes between dry and soaked conditions, with only slight variations in both surface deviation and volumetric gain. Notably, MC demonstrated a reduction in surface deviation after soaking (1.92 ± 0.59 mm to 1.29 ± 0.46 mm) and a marked decrease in volumetric gain (294.58 ± 88.78 mm^3^ to 202.64 ± 72.19 mm^3^).

Overall, hydration tended to increase the dimensional expansion and volumetric gain for most matrices, with FG6 and FG3 showing the most pronounced changes, while MC displayed the lowest expansion and the only reduction following soaking.

### 3.5 Correlation Between the Mean Pixel Echo Intensity (EI) and Volume Expansion In Vitro and Ex Vivo

Spearman's correlation analysis showed no statistically significant correlation between HFUS density and volume expansion. Weak correlations were observed between HFUS density and volume expansion measured in vitro (*r* = 0.29, *p* = 0.535), ex vivo dry (*r* = −0.21, *p* = 0.645), and ex vivo wet conditions (*r* = −0.32, *p* = 0.482). Scatterplots for the ex vivo baseline–dry and baseline–wet comparisons showed a similar pattern, with a negative slope of the regression line indicating that lower gray levels tended to be associated with greater expansion; however, these results were not statistically significant (Figure S3).

## 4 Discussion

The present study analyzed the behavior of collagen‐based soft tissue substitutes after hydration. This is based on a clinical observation of collagen matrices resulting in substantial swelling during surgery and within the first post‐operative week to an extent that is more distinct than observed with autogenous soft tissue grafts.

Both in vitro and ex vivo analyses predominantly revealed the following findings:

I. Fibro‐Gide 6 mm and Mucogain 5 mm demonstrated the highest absorption index and the greatest increase in weight over time.
II. These two matrices also exhibited the largest overall volumetric and mean expansion (mean Fibro‐Gide 6 mm 0.48 ± 0.06 mm and Mucogain 5 mm 0.53 ± 0.40 mm). Mucogain 5 mm showed a predominant occlusal increase, whereas Fibro‐Gide 6 mm expanded mainly laterally.
III. Mucogain 5 mm displayed the highest EI, which corresponds to the density of a material, while Volumax showed the lowest elasticity index based on SR measurements.
IV. The ex vivo analysis revealed that volume‐stable matrices are more prone to expansion when soaked with blood, even under a flap, whereas non‐volume stable matrices showed a tendency to collapse.

Collagen‐based matrices are increasingly used as alternatives to autogenous connective tissue grafts to reduce morbidity and surgical complexity (Thoma, Strauss et al. 2023; Yadav et al. 2025). Their clinical performance depends on biological integration and their early biomechanical response to blood and tissue‐fluid hydration (Bienz et al. 2023; Yao et al. 2008).

In the present study, volume stable matrices exhibited environment‐dependent behavior: they permitted substantial fluid uptake and hydration‐driven expansion under unconstrained in vitro conditions, while resisting compression, collapse, and shrinkage beneath a sutured flap ex vivo. Their ex vivo structural stability should therefore be interpreted as preservation of the hydrated scaffold volume under mechanical confinement rather than simple maintenance of the initial dry volume. These findings indicate that hydration‐driven expansion and resistance to compressive forces are complementary biomechanical properties of cross‐linked matrices. Understanding these responses is essential for surgical planning, as excessive expansion or insufficient resistance to compression may affect flap adaptation, wound stability, and the risk of postoperative dehiscence.

FG6 and MG5 demonstrated the highest absorption index (AI) and the greatest weight increase following hydration. This pronounced fluid uptake suggests a highly porous and hydrophilic matrix architecture. Such characteristics may facilitate rapid blood infiltration and clot stabilization, which are essential prerequisites for early vascularization and tissue integration of collagen scaffolds. Previous experimental and clinical studies have highlighted that porous collagen matrices can support early fibroblast migration and angiogenesis (Schmitt et al. 2016; Song et al. 2019; Yao et al. 2008). However, while high fluid absorption may be advantageous for biological integration, excessive hydration may also lead to significant dimensional changes that could compromise the predictability of soft tissue augmentation procedures and wound stability primarily during the early healing phase (up to suture removal).

Consistent with their high absorption capacity, FG6 and MG5 also showed the greatest volumetric expansion after hydration. Notably, the direction of expansion differed between the matrices, with MG5 predominantly expanding in the vertical direction, whereas FG6 exhibited mainly lateral expansion. These findings suggest that the internal architecture of the matrices may influence the pattern of dimensional change following hydration. From a clinical perspective, this observation may be relevant, as different expansion patterns could influence the final soft tissue contour. Vertical expansion may contribute to increased soft tissue thickness, whereas lateral expansion may affect the horizontal contour and tissue volume. However, excessive or uncontrolled swelling may also interfere with flap adaptation or lead to overcontouring of the augmented area (Pirc et al. 2025). Similar dimensional changes have been reported in previous investigations evaluating collagen matrices, highlighting that swelling behavior is an inherent property of many collagen‐based scaffolds (Thoma et al. 2014; Zeltner et al. 2017). These findings underline the importance of considering not only the volumetric stability of such matrices over time but also their immediate behavior after hydration. Consequently, the flap design needs to be adjusted according to the expected swelling occurring during the early phase postoperatively.

Ultrasound analysis further demonstrated differences in the structural characteristics of the tested matrices. MG5 exhibited the highest density, reflected by increased echo intensity, indicating a more compact internal architecture. In contrast, VC showed the weakest elasticity index based on strain ratio measurements, suggesting less flexibility of the material. Mechanical properties such as density and elasticity are likely to influence the clinical handling and performance of soft tissue substitutes. A denser scaffold may provide improved space‐maintaining capacity during the early healing phase, which is considered important for preserving augmented tissue volume (Herford et al. 2019). Moreover, the density of a substance may influence its propensity for hydration and its absorption characteristics. Conversely, matrices with greater elasticity may adapt more readily to surrounding tissues and mechanical forces. However, excessive elasticity may also reduce structural resistance and increase the risk of collapse under soft tissue pressure.

The ex vivo findings further highlighted the influence of collagen cross‐linking on matrix behavior following hydration. Volume‐stable matrices tended to expand when soaked with blood even when placed under a flap, whereas non‐cross‐linked matrices showed a tendency to collapse. This observation is in line with previous studies indicating that cross‐linking enhances the structural stability and enzymatic resistance of collagen‐based biomaterials (Rothamel et al. 2014). Increased stability may improve the ability of the matrix to maintain space during the early healing phase. However, cross‐linking may also alter the mechanical behavior of the scaffold and potentially affect cellular infiltration and remodeling dynamics.

In contrast, non‐cross‐linked matrices are generally characterized by faster degradation and remodeling, which may facilitate rapid integration with host tissues but may also compromise their ability to maintain volume during early healing (Schmitt et al. 2013). Clinical studies evaluating collagen matrices for soft tissue augmentation have reported favorable tissue integration but also variable degrees of volumetric reduction during the remodeling phase (Jepsen et al. 2026; Mayer et al. 2025). These findings highlight the inherent trade‐off between structural stability and biological remodeling in collagen‐based biomaterials.

In summary, the results of the present study indicate that hydration behavior, expansion characteristics, matrix density, and cross‐linking properties play a critical role in determining the early dimensional stability of collagen‐based soft tissue substitutes. In particular, FG6 and MG5 demonstrated pronounced fluid absorption and volumetric expansion, which may support early stabilization of the scaffold but may also introduce potential variability in the final augmented tissue contour. Consequently, careful consideration of the material‐specific properties of collagen matrices appears essential when selecting the surgical approach and the respective periosteal releasing incisions.

Finally, it should be emphasized that the present results were obtained under in vitro and ex vivo conditions. While these models provide valuable insights into the physical behavior of biomaterials, they cannot fully replicate the complex biological environment encountered in clinical situations. Factors such as vascularization, enzymatic degradation, mechanical loading, and host immune responses may substantially influence the remodeling and long‐term stability of collagen matrices. Furthermore, the ultrasonographic properties reported here reflect the matrices in their native state; once stabilized underneath a coronally advanced flap and subjected to the dynamic conditions of wound healing, their echogenicity, thickness, and elasticity are expected to change over time in ways that cannot be predicted from the present data alone. A critical limitation of the present study is the use of sodium citrate to prevent blood coagulation. Under clinical conditions, collagen matrices do not act merely as passive sponges for liquid blood; they interact with platelets and the active coagulation cascade, support fibrin polymerization, and become integrated within the developing clot. Consequently, the biomechanical behavior of a clot‐integrated matrix may differ substantially from the hydration‐related expansion measured under noncoagulating conditions. Anticoagulated blood was nevertheless selected to standardize fluid exposure and isolate the intrinsic absorption and swelling behavior of the tested matrices, thereby reducing variability associated with clotting kinetics, fibrin formation, and clot contraction. The present findings should therefore be interpreted as comparative measurements of material‐specific hydration behavior rather than as direct predictions of in vivo volume gain. Although this controlled approach improves intermaterial comparability, it reduces physiological validity and cannot reproduce the combined effects of clot formation, flap pressure, tissue perfusion, and early remodeling. Future investigations using active coagulation models are needed to determine how scaffold–clot integration modifies the magnitude and direction of volumetric changes observed clinically. The ex vivo findings should also be interpreted in light of the mechanical and hydrostatic characteristics of the model. Injection of blood beneath a closed, sutured flap may generate transient hydrostatic pressure and fluid pooling, producing a ballooning effect that cannot be fully distinguished from intrinsic matrix expansion. Therefore, the observed volumetric changes represent the combined effects of scaffold hydration, fluid displacement, and flap mechanics. Any manipulation of the sutures during blood delivery could additionally modify the initial flap tension and influence the subsequent measurements. Moreover, matrices with different initial thicknesses were not exposed to equivalent mechanical conditions: a 6‐mm matrix inherently produced greater baseline flap elevation and potentially greater flap tension than a 3‐mm matrix. Differences between thickness groups may consequently reflect variations in confinement and compressive loading in addition to material‐specific behavior. Future studies should preserve the sutured environment using microneedle delivery and quantify baseline flap tension or pressure to better isolate intrinsic matrix expansion.

## 5 Conclusion

Collagen‐based soft tissue substitutes demonstrated a distinct hydration and volumetric expansion following blood exposure. Volume‐stable matrices showed greater fluid absorption and volumetric rebound, resulting in greater initial dimensional stability compared with non‐volume stable matrices. However, their pronounced expansion highlights the importance of careful clinical handling to avoid excessive flap tension during flap closure and reduce the rate of wound dehiscence. Conversely, non–cross‐linked matrices exhibited lower expansion and a tendency toward collapse, which may favor early biological integration but may compromise initial volume maintenance. Understanding these material‐specific properties may help clinicians optimize matrix selection and surgical management to achieve more predictable outcomes in soft tissue augmentation.

## Author Contributions

Leonardo Mancini, Stephanie Wenk, and Daniel S. Thoma were involved in the conception and design of the study. Leonardo Mancini and Stephanie Wenk were involved in the collection of the data. Leonardo Mancini and Lorenzo Tavelli were involved in the statistical analysis and interpretation of the data. Leonardo Mancini, Stephanie Wenk, Lorenzo Tavelli, Ronald E. Jung, Nadja Naenni, and Daniel S. Thoma were involved in the final drafting of the work. All the authors were involved in the critical manuscript review and are accountable for all aspects of the work.

## Conflicts of Interest

The authors do not have any financial interests, either directly or indirectly, in the products or information listed in the paper.

## Supporting information


**Figure S1:** (A) Microscope slide equipped with two scanning pins. (B) Baseline condition during scanning. (C) Matrix after soaking. (D) Scanning performed after completion of the soaking process.

**Figure S2:** This graph shows mean values (±SD). The overall level of the weight (mg) depends on the group and different patterns (slopes of increment) over the period. Dry (T0), 30 s (T1), 2 h (T2), 4 h (T4), 6 h (T6), and 12 h (T12). (FG6) Fibro‐Gide 6 mm; (FG3) Fibro‐Gide 3 mm; (MG5) Mucogain 5 mm; (MG3) Mucogain 3 mm; (VC), Volumax; (CG2) Collagen Graft 2; (MC) Mucograft.

**Figure S3:** Spearman's correlation analysis indicating a trend toward a negative association between density and volume expansion. (FG6) Fibro‐Gide 6 mm; (FG3) Fibro‐Gide 3 mm; (MG5) Mucogain 5 mm; (MG3) Mucogai 3 mm; (VC) Volumax; (CG2) Collagen Graft 2; (MC) Mucograft.

**Table S1:** Absorption index (AI) of the different collagen matrices at various time points, showing changes according to sample type, thickness, and soaking time. Dry (T0), 30 seconds (T1), 2 hours (T2), 4 hours (T4), 6 hours (T6) and 12 hours (T12); (FG6) Fibro‐Gide® 6mm; (FG3) Fibro‐Gide® 3mm; (MG5) Mucogain® 5mm; (MG3) Mucogain® 3mm; (VC) Volumax®; (CG2) Collagen Graft 2®; (MC) Mucograft®.
**Table S2:** mean grey levels, skewness and kurtosis of the different substitutes mean and standard deviation. GL grey levels, SD standard deviation. (FG6) Fibro‐Gide® 6mm; (FG3) Fibro‐Gide® 3mm; (MG5) Mucogain® 5mm; (MG3) Mucogain® 3mm; (VC) Volumax®; (CG2) Collagen Graft 2®; (MC) Mucograft®.

## Data Availability

The data that support the findings of this study are available from the corresponding author upon reasonable request.
