# Supplementary material for: Collagen Substitutes in Oral Soft Tissue Augmentation: An In Vitro–Ex Vivo Study on Expansion and Elasticity
Source: Clin Exp Dent Res. 2026 Sep 8;12(5):e70448. doi: 10.1002/cre2.70448 (PMC13552866; doi:10.1002/cre2.70448)
Supplement: Supplementary file 1 [file CRE2-12-e70448-s004.docx]

| Group | T1 (mean ± SD) | T2 (mean ± SD) | T4 (mean ± SD) | T6 (mean ±SD) | T12 (mean ± SD) |
| --- | --- | --- | --- | --- | --- |
| FG6 | 17.01±0.63mg | 17.67±0.90mg | 18.05±0.87mg | 18.18±0.71mg | 17.96±0.72mg |
| FG3 | 18.28±2.31mg | 18.53±1.76mg | 19.56±1.78mg | 18.95±1.43mg | 18.57±1.15mg |
| MG5 | 12.06±1.98mg | 16.78±2.79mg | 18.76±2.70mg | 19.40±2.22mg | 20.33±1.55mg |
| MG3 | 17.15±2.74mg | 19.72±1.66mg | 20.67±1.77mg | 21.19±1.71mg | 21.98±1.28mg |
| VC | 4.14±0.47mg | 6.66±0.44mg | 9.04±0.34mg | 10.19±0.57mg | 11.33±1.78mg |
| CG2 | 12.01±0.73mg | 13.16±0.29mg | 13.32±0.45mg | 13.83±0.52mg | 14.04±0.75mg |
| MC | 12.02±3.91mg | 15.09±2.91mg | 15.61±3.01mg | 15.72±2.41mg | 16.40±3.10mg |

**Supplementary Table 1.** Absorption index (AI) of the different collagen matrices at various time points, showing changes according to sample type, thickness, and soaking time. Dry (T0), 30 seconds (T1), 2 hours (T2), 4 hours (T4), 6 hours (T6) and 12 hours (T12); (FG6) Fibro-Gide®6mm; (FG3) Fibro-Gide® 3mm; (MG5) Mucogain® 5mm; (MG3) Mucogain® 3mm; (VC), Volumax®; (CG2) Collagen Graft 2®; (MC) Mucograft®.

| Mean±SD | FG6 | FG3 | MG5 | MG3 | VC | CG2 | MC |
| --- | --- | --- | --- | --- | --- | --- | --- |
| Mean grey level (GL) | 62±5.9 | 70.32±19.3 | 105.36±6.2 | 90.60±36.4 | 86.57±9.06 | 85.44±11.19 | 73.05±13.9 |
| Skewness (GL) | 1.51±0.28 | 1.63±0.59 | 0.29±0.09 | 0.88±1.16 | 0.63±0.17 | 0.79±0.29 | 1.26±0.31 |
| Kurtosis (GL) | 3.78±1.47 | 5.46±3.92 | -0.57±0.13 | 2.15±4.33 | -1.11±0.22 | -0.39±0.30 | 1.29±1.24 |

**Supplementary Table 2.** Mean grey levels, skewness and kurtosis of the different substitutes mean and standard deviation. GL grey levels, SD standard deviation. (FG6) Fibro-Gide®6mm; (FG3) Fibro-Gide® 3mm; (MG5) Mucogain® 5mm; (MG3) Mucogain® 3mm; (VC), Volumax®; (CG2) Collagen Graft 2®; (MC) Mucograft®.
